# Lessons learned from the PMTCT program in Swaziland: challenges with accepting lifelong ART for pregnant and lactating women – a qualitative study

**DOI:** 10.1186/s12889-016-3767-5

**Published:** 2016-10-24

**Authors:** Leila Katirayi, Caspian Chouraya, Kwashie Kudiabor, Mohammed Ali Mahdi, Mary Pat Kieffer, Karen Marie Moland, Thorkild Tylleskar

**Affiliations:** 1 Elizabeth Glaser Pediatric AIDS Foundation, 1140 Connecticut Ave NW, Suite 200, Washington DC, 20036 USA; 2 Elizabeth Glaser Pediatric AIDS Foundation, Mbabane, Swaziland; 3 Center for International Health, University of Bergen, Bergen, Norway

## Abstract

**Background:**

Swaziland has one of the highest HIV prevalence rates in sub-Saharan Africa, 26 % of the adult population is infected with HIV. The prevalence is highest among pregnant women, at 41.1 %. According to Swaziland’s prevention of mother-to-child transmission (PMTCT) guidelines, approximately 50 % of pregnant women are eligible for antiretroviral therapy (ART) by CD4 criteria (<350 cells/ml). Studies have shown that most mother-to-child transmission and postnatal deaths occur among women who are eligible for ART. Therefore, ensuring that ART eligible women are initiated on ART is critical for PMTCT and for mother and baby survival. This study provides insight into the challenges of lifelong ART initiation among pregnant women under Option A in Swaziland. We believe that these challenges and lessons learned from initiating women on lifelong ART under Option A are relevant and important to consider during implementation of Option B+.

**Methods:**

HIV-positive, treatment-eligible, postpartum women and nurses were recruited within maternal and child health (MCH) units using convenience and purposive sampling. Participants came from both urban and rural areas. Focus group discussions (FGDs) and structured interviews using a short answer questionnaire were conducted to gain an understanding of the challenges experienced when initiating lifelong ART. Seven FGDs (of 5–11 participants) were conducted, four FGDs with nurses, two FGDs with women who initiated ART, and one FGD with women who did not initiate ART. A total of 83 interviews were conducted; 50 with women who initiated ART and 33 with women who did not initiate. Data collection with the women was conducted in the local language of SiSwati and data collection with the nurses was done in English. FGDs were audio-recorded and simultaneously transcribed and translated into English. Analysis was conducted using thematic analysis. Transcripts were coded by two researchers in the qualitative software program MAXqda v.10. Thematic findings were illustrated using verbatim quotes which were selected on the basis of being representative of a specific theme. The short-answer interview questionnaire included specific questions about the different steps in the woman’s experience initiating ART; therefore the responses for each question were analyzed separately.

**Results:**

Findings from the study highlight women feeling overwhelmed by the lifetime commitment of ART, feeling “healthy” when asked to initiate ART, preference for short-course prophylaxis and fear of side effects (body changes). Also, the preference for nurses to determine on an individual basis the number of counseling appointments a woman needs before initiating ART, more information about HIV and ART needed at the community level, and the need to educate men about HIV and ART.

**Conclusion:**

Women face a myriad of challenges initiating lifelong ART. Understanding women’s concerns will aid in developing effective counseling messages, designing appropriate counseling structures, understanding where additional support is needed in the process of initiating ART, and knowing who to target for community level messages.

## Background

In Africa, mother-to-child human immunodeficiency virus (HIV) transmission (MTCT) is the largest source of infection in children under 10 years old. Without any preventive intervention, the risk of MTCT is estimated to range from 25 to 40 % in low income countries, where breastfeeding is recommended to HIV-positive women [1]. Prevention of MTCT requires women to access a sequential cascade of services beginning with accessing the antenatal care clinic (ANC) and testing for HIV. For those who test HIV positive, additional steps include determining eligibility for antiretroviral treatment (ART) by performing a CD4 count or applying WHO clinical staging (prior to universal ART for all pregnant women being recommended), initiating ART, adhering to ART, accessing the post-natal care (PNC) services, testing the infant for HIV and initiating the infant on treatment if necessary [2]. Ensuring that HIV-positive pregnant women receive the full cascade of PMTCT services is critical to ensuring a generation free of HIV and acquired immunodeficiency syndrome (AIDS).

Swaziland’s HIV prevalence rate of 41 % is among the highest in the world among pregnant women [3]. With an estimated 34,571 deliveries in 2015, ensuring that HIV positive pregnant women receive PMTCT services is critical to reducing the number of HIV-positive women who transmit the virus to their children [4] and make progress toward a generation free of HIV.

In 2010, Swaziland began implementing the World Health Organization (WHO)’s Option A guidelines, which recommend that HIV-positive women with a CD4 count less than 350 cells/mm or WHO clinical stage III/IV are offered lifelong ART, while those with CD4 above 350 cells/mm and are stage I/II are offered short-course prophylaxis. Short-course prophylaxis includes zidovudine from 14 weeks of pregnancy until labor and delivery; during labor women receive single-dose nevirapine with lamivudine/zidovudine, continuing the lamivudine/zidovudine through 7 days postpartum, and infants receive daily nevirapine until cessation of breastfeeding [5].

In 2013, the WHO guidelines recommended that all HIV-infected pregnant and lactating women, regardless of CD4 cell count or clinical status, initiate ART during pregnancy, which should be continued lifelong (Option B+) in high prevalence settings such as Swaziland [6]. These revised guidelines were a significant change from the previous guidelines, as women with CD4 counts above 350 who may be asymptomatic and perceive themselves as healthy are now offered lifelong ART instead of short-course prophylaxis. In October 2014, Swaziland initiated Option B+ as the national PMTCT strategy which at the time of writing is in the process of national roll-out.

The overall acceptance and uptake of lifelong ART among pregnant and lactating women in Swaziland has been a challenge. In 2009, only about 50 % of eligible women were initiated on ART [7]. However, by 2013, due to intensified client education and improved guidelines 72 % of eligible women had initiated ART [8]. Previous studies have attributed failure or delay of ART initiation to a variety of factors, including stigma, lack of disclosure, the need for partner support, food insecurity, transportation time and cost, negative perceptions of antiretroviral drugs (ARVs), trust in faith healing and traditional medicine, cost of treatment, lack of drugs availability, staff shortages and poor treatment of clients by health care workers [9–13].

Minimal research has been done, however, to understand the challenges related to ART initiation among pregnant women beginning a lifelong commitment to treatment, whether initiation is for maternal health under Option A or more broadly for maternal health and prevention of MTCT under Option B+. This study provides insight into the challenges of lifelong ART initiation among pregnant women under Option A in Swaziland. We believe that these challenges and lessons learned from initiating women on lifelong ART under Option A are relevant and important to consider during implementation of Option B+.

## Methods

### Study sites

The study was conducted in the four regions of Swaziland: Shiselweni, Lubombo, Hhohho, and Manzini. Study sites with the highest number of annual deliveries in both urban and rural locations were purposively selected.

### Study participants

The study participants included nurses and HIV-positive, postpartum women who were eligible for ART during their pregnancy under Option A (CD <350 and/or WHO Stage III/IV disease). The study recruited both women who did and women who did not initiate ART during their pregnancy. Nurses who participated in the study were employed in the maternal and child health unit for a minimum of 1 year.

### Data collection

Data with the HIV-positive, postpartum women were collected through individual interviews and focus group discussions (FGDs) in the local language of SiSwati and FGDs with nurses were conducted in English. The study originated after review of routine program M&E data by the Elizabeth Glaser Pediatric AIDS Foundation (EGPAF) which showed that HIV-positive ART-eligible pregnant women were not initiating ART. The study was designed to answer the main question of why women were choosing not to initiate ART when they understood that it would prolong their lives and potentially prevent transmission to their unborn infants. A free listing of potential reasons was made by the study team and health care providers were consulted to ensure all critical topic areas were included. The data collection tools were then drafted and piloted at non-study sites to ensure that questions were correctly understood and to estimate the time to complete the interviews and FGDs.

Short-answer questionnaires were used to conduct individual interviews with women in the maternity ward after they had delivered their child and before being discharged. Since many women do not return for post-natal care (especially those who did not initiate ART), interviewing women in the maternity ward provided an opportunity to interview more women who did not initiate ART. Considering that these women had recently given birth, the study team did not feel it was appropriate to administer an in-depth interview and instead opted for a short answer questionnaire which would take less time to answer the priority research questions. The short answer questionnaire included a combination of open- and closed-ended questions to understand women’s individual trajectories from learning their HIV status to deciding whether or not to initiate ART, with a focus on potential barriers to initiation of ART. The closed-ended questions included yes/no questions, multiple choice and Likert scale questions. The closed-ended questions were primarily used to gather demographic data, information about when women learned their HIV status, if/when they initiated ART, whether ART was provided at their facility or they had to be referred and who they consulted before initiating ART. The Likert question asked on a scale of 1–5 how difficult it was to initiate ART. The opened ended questions were used to gather information about messages they heard at the facility, what information was unclear, why they decided to initiate or not initiate ART, what challenges they faced initiating ART and what were the anticipated challenges with continuing medication and returning to the facility. Convenience sampling was used to select participants in the maternity ward. From July to September 2011 all eligible women at the selected study sites were invited to participate in the study. Nurses were trained by the Elizabeth Glaser Pediatric AIDS Foundation (EGPAF) staff to identify eligible women, obtain written informed consent, and conduct interviews.

Women who participated in the FGDs were recruited from the child welfare clinics when they returned for the child’s immunization visit at 6 or 10 weeks. These time periods were selected to reduce recall bias and due to high drop-out rate for the third immunization visit at 14 weeks. Post-delivery is a common drop-off point in the PMTCT cascade therefore the study sought to gather the perspectives from multiple time points (immediately following delivery, 6 and 10 weeks). Convenience sampling was also used to recruit women for the FGDs. Between January to March 2012 all eligible women who attended the child welfare clinics at the selected sites were invited to participate in the FGDs. Nurses were trained on participant eligibility criteria and referred all eligible women to onsite RAs who enrolled them in the study. Those who participated in the FGDs were told a day and time to return to the facility to participate in the FGD. The FGD used a semi-structured guide with open-ended questions to facilitate a discussion about the barriers and facilitators faced by women when deciding to initiate ART under Option A. All FGDs were conducted by EGPAF staff trained in qualitative data collection. All FGDs had 5–11 participants, were conducted with one moderator and one note taker, and were audio-recorded.

Purposive sampling was used to recruit nurses for FGDs between January to March 2012. Nurses were recruited for FGDs by EGPAF regional PMTCT coordinators in collaboration with the regional clinic supervisors. Given the small number of nurses who provide services at these facilities, all eligible nurses providing services in each of the four regions of Swaziland were invited to participate, and this did not exceed the maximum number of FGD participants. The regional coordinator informed the participants about the time and day the FGD would be conducted. FGDs with nurses were similarly conducted using a semi-structured guide with open-ended questions focused on barriers and facilitators to women initiating ART, as perceived by nurses. The nurses who participated in the FGDs were not the same nurses who interviewed patients, as the FGD participants were recruited from the maternal and child health units, and nurses who conducted interviews were based in the maternity ward.

Prior to implementation all study materials were reviewed and approved by the Swaziland Scientific and Ethics Committee. Individuals who participated in the interviews provided written informed consent, while FGD participants provided verbal consent. All efforts were made to protect participant privacy by limiting collection of  any identifiable information, limiting access of study data to study staff, and using study ID numbers instead of names when conducting FGDs.

### Data analysis

Audio-recordings of the interviews and FGDs with the women were transcribed and translated into English simultaneously. Data were analyzed using thematic analysis [14]. After transcription, a master list of all findings was assembled, and codes were determined when a finding was seen to reoccur throughout the data and was relevant to the research questions. Related codes were grouped into overarching themes. The codes and themes were used to create a coding framework. The FGDs were coded in the qualitative software program MAXqda v.10 by two members of the study team who designed the study. Thematic findings were illustrated using verbatim quotes which were selected on the basis of being representative of a specific theme.

 The short answer questionnaire included specific questions about the different steps in the woman’s experience initiating ART; therefore it was more appropriate to analyze the responses for each question separately. Responses for each question were compiled and examined for similaries and differences among the responses and compared to the FGD data. 

## Results

A total of 83 individual interviews were conducted; 50 with women who chose to initiate ART and 33 with women who chose not to initiate ART. Seven FGDs were carried out with 59 participants; four FGDs with nurses, two FGDs with women who initiated ART, and one FGD with women who did not initiate ART. To indicate which results were more frequently discussed, the results are presented in the order of least frequently discussed to most frequently discussed within the two themes. Study results are highlighted in Table 1.

**Table 1:** Key study findings

Challenges accepting lifelong ART	
Challenge accepting ART when feeling “healthy”	• Women struggled with to accept ART when they felt healthy because ART is associated with being very ill or having a low CD4 count.
Preference for short-course prophylaxis	• Women preferred the short-course prophylaxis so that they could avoid disclosure and tell their partner or family that the drugs were related to the pregnancy
Overwhelmed by lifetime commitment	• Most women were familiar with the concept of developing resistance, which caused some to delay initiation until they felt “ready” to commit for life
More information needed on ART	
Fear of side effects	• The fear of side effects was often related to deformities and changing physical appearance. Nurses believed this was associated with a drug no longer in use (Stavudine)
Number of clinic appointments	• Nurses reported considerable variation in the number of adherence counseling appointments a woman needed and advocated for the nurse's discretion to determine the number of adherence counseling sessions on an individual basis
More information needed at the community level	• Nurses reported being too busy at the facility to provide education sessions in the community (which had previously been provided)
Educating men about HIV and ART	• Women reported their partners lacking information about HIV and ART and becoming abusive at the mention of either topic

The majority of women who participated in the interviews were between 21 and 30 years of age (Table 2). Interview participants who did initiate ART were more commonly single while those who did not initiate ART were more commonly married. The majority of FGD participants were married. For the FGD participants, the most common age was between 26-30. Across the interviews and FGDs, most women were Protestant, and the most common educate level was secondary, except for the FGD participants who did not initiate ART, whose common education level was tied between secondary and primary. The majority of nurses were between the ages of 36-45, employed as a staff nurse and had been employed for 1-5 years (Table 3). Detail about nurse designations is provided in Table 4.

**Table 2:** Demographic characteristics for postpartum women participants

	Individual interviews		Focus group discussions	
	Initiated ART *N* = 50 *n* (%)	Did not initiate ART *N* = 33 *n* (%)	Initiated ART *N* = 16 *n* (%)	Did not initiate ART *N* = 6 *n* (%)
Age (years)				
16–20	5 (10 %)	5 (15 %)	0	0
21–25	19 (38 %)	10 (30 %)	5 (31 %)	1 (17 %)
26–30	12 (24 %)	11 (33 %)	5 (31 %)	4 (67 %)
31–35	10 (20 %)	6 (18 %)	5 (31 %)	1 (17 %)
36–40	2 (4 %)	0	1 (6 %)	0
41–44	2 (4 %)	1 (3 %)	0	0
Marital status				
Married/living with partner	23 (46 %)	18 (54 %)	11 (69 %)	4 (66 %)
Single	27 (54 %)	15 (45 %)	5 (31 %)	2 (33 %)
Education				
None	2 (4 %)	1 (3 %)	0	0
Primary	14 (28 %)	9 (27 %)	5 (31 %)	3 (50 %)
Secondary	31 (62 %)	22 (67 %)	10 (63 %)	3 (50 %)
Tertiary	3 (6 %)	1 (3 %)	1 (6 %)	0
Religion				
None	0	2 (6 %)	0	0
Catholic	2 (4 %)	0	2 (13 %)	0
Protestant	39 (78 %)	27 (83 %)	14 (87 %)	6 (100 %)
Islam	0	0	0	0
Other	9 (18 %)	4 (12 %)	0	0

**Table 3:** Demographic characteristics for the participating nurses

	Nurses *N* = 35 *n* (%)
Age (in years)	
26–30	6 (19)
31–35	6 (19)
36–40	9 (29)
41–45	9 (29)
46+	1 (3)
Level of qualification	
General nurse	4 (13)
Nurse midwife	27 (87)
Current designation	
Nurse midwife	6 (19)
Senior nurse	4 (13)
“Sister”	2 (6)
Staff nurse	19 (61)
Years in current position	
1–5	14 (45)
6–10	8 (26)
11–15	5 (16)
16–21	4 (13)

**Table 4:** Nurse designations

Nurse designation (lowest to highest ranking position)	Role of nurse
Nursing Assistant	This level nurse as depicted by the title assists the higher level nurses. They have very limited duties such as providing childhood immunization; dispensary and they are not allowed to prescribe.
State registered Nurse (Staff nurse/General nurse)	This level nurse does everything the midwives do except that they do not conduct ANC, maternity/delivery services.
Nurse midwife	This level nurse performs ANC services, delivery, post-natal care, and other services related to maternal child health and curative services. They are usually the nurse-in-charge/senior nurse of the facility when there are no doctors present.
Senior nurse	This is more of the role than qualification. This person is usually the most senior midwife at the facility where there are multiple individuals with the same qualification In most cases, this person is the nurse-in charge and also oversee the day-to-day running of the facility just like the nurse-in charge or in the absence of a nurse-in charge.
Nurse-in charge (Sister)	This is the person in charge of the local health facility. They oversee the day-to-day running of the facility just like the senior nurse.

### Challenges accepting lifelong ART

The most frequently cited challenges accepting lifelong ART were feeling “healthy” when asked to initiate ART, being overwhelmed by the lifetime commitment of drugs, and preference for short-course prophylaxis.

#### Feeling “healthy” when asked to initiate ART

Both women and nurses frequently mentioned the belief that ART is for very sick people and that it can be very difficult for a woman to accept ART when she is feeling healthy.

*Sometimes you can even ask if the nurses are really serious or if they are just joking when they tell you that you are now eligible for HIV medicine when in actual fact you feel healthy and haven’t fallen sick (Woman).*

Nurses discussed the challenge of women believing that ART is only for the very ill.

*Sometime you find that these women come knowing their HIV status while others come with unknown HIV status. Those who come up [test] positive, they tend not to go for ART because they believe ART is for those people, say those whose CD4 are very low or those who are almost sick so for them they don’t feel the need to start the ART because …eh…eh…their coming to the facility is to start ANC so starting treatment when they are not sick, no they…they… they don’t feel like [starting ART] (Nurse).*

Because these women are feeling healthy and are not experiencing any symptoms it becomes difficult for them to justify starting ART.

*If for instance, you were to tell me right now that because I have ‘brackets’ I need to put on casts to strengthen my legs I won’t agree because I am comfortable with the way I am. So even with these women it’s the same thing. I come here healthy to do my ANC and then all of a sudden you are telling me I’m sick. I have to be on ART. I think that’s the other problem. These women come here feeling very healthy and now they are going back home with a sack of tablets (Nurse).*

Nurses discussed how it’s more challenging for those who are just learning their HIV status to initiate ART.

*Those who are pregnant and have found out about their HIV positive status during the pregnancy, are usually the most difficult to initiate. But the one who fell pregnant knowing her status is generally easier to initiate. So I think we should strengthen teachings on HTC and encourage women to make sure that they know their HIV status before falling because, like I said it’s easier for those who know their HIV status than this one (coughs) who has just come and is newly diagnosed HIV positive (Nurse).*

#### Preference for short course prophylaxis to avoid stigma

The nurses discussed women wanting short course prophylaxis, especially younger women and those who had been pregnant before and were familiar with the short course prophylaxis.

*I wanted to comment on the issue of those women who were pregnant before, and were given intrapartum dose* 1 *and gave birth to a HIV negative baby, and now on her second pregnancy you tell her that she now has to initiate ART. Most of these women find this very hard to take because they do not understand why they cannot be given the short course instead of the lifetime commitment. There is a lot of resistance from these women in most instances, but with on-going counselling you are able to convince some (Nurse).*

Nurses discussed that women prefer the short course prophylaxis to ART to avoid disclosure to their partner by claiming the drugs are for the pregnancy.

*…some of them say that they haven’t disclosed to their partners so they say it’s difficult to start ART especially if you haven’t disclosed your HIV status. They say it’s better [to take drugs] during the gestation period since they can just take the drugs as if they are drugs taken during pregnancy. But then if she gives birth then it’s a problem because she has to stop taking the drugs since she has not disclosed to the partner (Nurse).*

Women also preferred the prophylaxis because they were able to receive medication in ANC and avoid stigma associated with the ART dispensary (when ART is not available in ANC).

#### Overwhelmed by lifetime commitment of drugs

Fear of commitment to lifetime therapy was mentioned frequently by nurses and women.

*I was just scared because I had heard them say that you have to take drugs for life and I was traumatised that I would have to drink them for the rest of my life…must I agree to this or not? I just couldn’t believe it such that I even cried when they told me that I would have to start taking the medication” (Woman).*

Women frequently discussed the use of drugs and concoctions to raise the CD4 count to avoid initiating lifetime therapy.

*The father of my baby said we can wait a little and use bio-plus (*a popular tonic in Southern Africa*) to boost CD4 instead of taking the medication spontaneously because the medication is lifetime bidding (Woman).*

Some women were not ready to make a lifetime commitment to ART. Nurses discussed some women throwing away the medication or delaying initiation after leaving the facility until they felt ready.

*I once heard that some women hide the pills when they reach home and with the hope that one day they will have the motivation to start taking them. It is said that they plan to keep them hidden for quite some time because they are told that once you start taking them it should be a lifetime commitment (Nurse).*

One of the concerns of lifelong ART was a fear of failure to adhere to ART and of developing resistance. The women also expressed concerns about potential stock-outs at the facility limiting their access to ART and thereby putting them at risk for developing resistance.

*(Women say) I don’t see myself being able to take the medication for the rest of my life because I don’t trust myself I will be able to follow all your instructions (Nurse).*

To help patients accept the lifelong commitment of ART, one nurse discussed comparing HIV to other chronic diseases that require lifelong medication.

*…what I usually tell them is that HIV is not the only illness where you have to take chronic medication. Think about diabetes, hypertension, epilepsy, asthma, heart disease and other stuff, so it’s the same with HIV! (Nurse).*

### More information needed on ART

The most frequently cited challenges regarding the need for more information about ART included: fear of side effects, the number of clinic appointments before initiating ART, needing more information about HIV and ART at the community level, and educating men about HIV and ART.

#### Fear of side effects

One of the barriers most frequently raised by women was fear of side effects caused by ART. The main side effects mentioned by women were: bursting liver, deformed body, shrinking buttocks, body rashes, developing humps, and looking like a man.

*I once had a patient who was adamant that she didn’t want to enroll on ART because she didn’t want to be deformed and be like ‘a man’… She also gave me an example of some people that she knows who have those side effects. She said some of those people were once pretty but now they are a far cry from their former self” (Nurse).*

Nurses believed that the side effects linked to deformity were most likely associated with stavudine, an ARV that is no longer in use. Throughout the FGDs both the women and nurses raised the topic that there needs to be more information about ART provided to women, specifically on the topic of side effects.

#### Number of clinic appointments before initiating lifelong ART

Some women and nurses felt that fewer appointments before initiating ART would decrease loss to follow-up, avoid expensive transportation costs and save time. Others felt that it was incredibly important to have multiple counseling appointments prior to ART initiation and have time to absorb their HIV-positive diagnosis before starting ART.

*Some women feel there are too many (adherence appointments). But women are different. Adherence counselling is very important (Nurse).*

Some nurses suggested flexibility regarding the number of pre-ART initiation appointments and allowing nurses the discretion to determine how many appointments each woman needs.

*One of the few issues that we have to take seriously because if we prescribe two sessions for all adherences, we are risking downfall in terms of technical outcomes… while we fast track pregnant, positive women but also try to evaluate individuals﻿;﻿ really is this person really going to be adherent? Do we still need more counselling sessions or not? The appointment date; let’s make it maybe closer, let’s not give one month supply. Let’s give it two weeks supply and have another adherence session in two weeks’ time. So I think it is important that we do individual evaluations and let’s not say two sessions and that’s it (Nurse).*

#### More information needed at the community level

Nurses discussed the need for additional information about HIV and ART to be shared at the community level. Some reported that additional community education could help decrease stigma.

*People do not know the importance of [HIV] testing and knowing your status. The people do not know, so they are not motivated to request that service. We have to do community mobilisation and health talks on HTC, and teach people and be ready to inform them about the current statistics. We need to tell people how HIV is linked to some of the illnesses currently prevailing in Swaziland. Therefore, from that stand point, everyone has to have information about HIV and be encouraged to test so that stigma can be curbed (Nurse).*

Nurses discussed being too busy at the facility to be able to provide health education at the community level.

*Back in the day nursing was not just limited to curative services and because of that we have the current disease burden because people are not educated. Back then as a nurse, you had to go to every community meeting to give a lesson on health. But because we now focus on curative interventions we are having these challenges (Nurse).*

#### Educating men about HIV and ART

Both nurses and women discussed the need for more efforts to engage and educate men. FGD discussions highlighted men’s fear of HIV and ART. When women visit the health facility for ANC appointments they are educated about HIV and ART, but the men do not have the same opportunity. Men’s lack of knowledge creates challenges for women. Women described informing male partners of their HIV-positive status, asking their partners to test and male partners becoming angry and sometimes violent.

*When I tried to convey the message of initiating ART he just became impossible. Even if I give him the card meant for our partners to come to the facility, he just acts wildly and threatens to punch me. When I told him I have since tested and I knew my status he just said keep that to you. I will know mine when my time has come. As for now I’m not even interested in knowing yours (Woman).*

Nurses and women discussed how men’s attitude toward HIV and ART discourages their female partners from testing and initiating ART. They may try to talk them out of initiating ART or confuse them with false information.

*Most often you find that men are misinformed about issues and end up confusing women who are about to start ART. So as part of improving our system we have to find a way to engage men (Nurse).*

The suggestions often included going to places that men frequent regularly, such as community meetings, churches and dip tanks and providing educational messages about HIV and ART.

*I’ll talk about something I came across in my own community. There were some improvements last year. There is an increase in male involvement.* (PSI organized a “Know Your HIV Status” Campaign targeted at men who brought their cattle to the dip tanks)*. So, the men would come and say, “I was at the dip tank and we got tested,”… and those men are different since they know their status… We still have some of them who come and retest. This shows that they really got the message about testing during the campaign (at the dip tank). So I think we have to involve communities* (Nurse).

## Discussion

These results identify challenges and barriers faced by pregnant and postpartum women when deciding to initiate ART under Option A, where only women with more advanced disease had ART recommended to them. Option B+ also requires a lifelong commitment to ART, therefore understanding the barriers women experienced under Option A can help to strengthen the acceptability of Option B+. These findings highlight the challenges of accepting lifelong ART and the need for more counseling during ART initiation.

### Challenges accepting lifelong ART

Many factors contribute to the challenge of accepting and initiating lifelong ART, including: the fear of developing resistance; disclosing one’s HIV status or dealing with a lifetime of hiding medications, and accepting and acknowledging one’s HIV status daily by taking medication.

Both women and nurses discussed the challenge of pregnant women initiating ART when they feel healthy despite having a low CD4 cell count. Under Option B+, the requirement for a woman to have a low CD4 count or advanced disease symptoms is removed and the short-course prophylaxis option is no longer available; therefore, more women who feel healthy will be offered ART. During the piloting of Option B+ in Swaziland, ART initiation rates were significantly lower among women with a high CD4 count: 59 % of women with CD4>350 initiated ART before 32 weeks gestation, compared with 80 % of women with CD4<350 [15].

Women who may have known their HIV status previously or anticipated that they may be HIV-positive may require fewer counseling sessions prior to initiating ART, while those struggling to accept their new HIV status and need to initiate ART may require more sessions. These women may need time to accept their HIV status, to disclose to a partner or family and be emotionally prepared to commit to lifelong ART. A study in Swaziland found that those who knew their HIV status before attending ANC were more likely to begin ART compared with those who were newly diagnosed during their ANC visit [15]. Research has also shown that loss to follow-up (LTFU) is greatest in the first 3 months after women are given ART under Option B+ when using the same day initiation approach [16]. Providing the opportunity for women to receive additional counseling sessions before initiating ART, especially among those newly diagnosed as HIV-positive, may reduce LTFU. In addition, a study on Option B+ in Malawi found that facilities that offered additional adherence counseling beyond the required national guidelines had lower rates of early LTFU [16]. Some women may require additional counseling due to personal circumstances such as an unanticipated HIV diagnosis, having a support structure which allows for disclosure and being knowledgeable about HIV and ART. Previous research has stressed the importance of understanding the variability in personal circumstances when it comes to counseling regarding ART and adherence [17]. Nurses in this study advocated for the ability to decide how many counseling appointments women need on an individual basis. This is an important factor to consider with Swaziland’s continued roll-out of Option B+.

Fear of developing ART resistance is a commonly cited barrier to treatment [18]. Study participants were aware that good adherence was necessary to avoid developing resistance. This awareness, however, also presented as a barrier for initiating ART, as some patients delayed initiation until they felt “ready to adhere for life.” In addition to the fear of not being able to be adherent and developing resistance, some participants believed that if they stopped taking ART at any point they would die. This concern has also been documented in other studies [19].

To help patients accept lifelong medication, some of the nurses discussed comparing HIV/AIDS to other chronic diseases which require lifelong medication, such as diabetes. They believed that these kinds of comparisons helped women to accept lifelong medication easier by not seeming so “different” from others. This approach could help mitigate the fear some patients have that being HIV-positive makes them different from other people [20].

Requesting the short-course prophylaxis was one way women attempted to avoid the commitment to lifelong ART and still provide protection to their unborn child. Strengthening the counseling messages to ensure that women understand how lifelong ART also benefits their health and partner’s health in addition to their child’s health is important [21]. The new messaging around Option B+ will be critical, as the previous messaging stated that ART is only for those with a CD4 count greater than 350 [15].

To avoid initiating lifelong ART, study participants discussed using drugs, herbs, and concoctions to raise the CD4 count above 350. Other studies have also cited the use of herbs and plants to raise CD4 counts [20]. These findings illuminate the lengths that some individuals are willing to go to avoid initiating ART. These fears and concerns about ART will continue unless they are dealt with directly through improved counseling and messaging.

### More information about ART

The results of this study highlight a clear need for sharing more information about ART, including its benefits and side effects, for the general population and for women at health facilities. The challenge of side effects has been frequently discussed in the literature [18, 22] and was also documented in this study. The descriptions of side effects in the reviewed literature focus on discomfort, such as headache, diarrhea, weight gain, dizziness, nightmares, nausea, pain and fatigue [18] while the side effects discussed in our study focus on changing physical features and deformities; study participants often mentioned “becoming ugly, looking like a man, losing the shape of one’s behind,” etc. Fear of treatment-induced body changes has also been discussed in a qualitative study from Zambia [20]. Nurses explained that such side effects are associated with ART and demonstrate the fear of undesired disclosure and potential stigma [23]. The nurses in this study mentioned that the side effects associated with disfiguration were often the result of stavudine, an ARV drug that is no longer in use. However, the discontinuation of this drug has not been discussed with the women or community at large. Information about the new drugs being used with fewer side effects under Option B+ needs to be communicated to the public.

Other research has cited the need for providing more information about PMTCT to the general public [24]. During the FGDs, nurses discussed that due to time constraints, they were often unable to spend adequate time in the community providing health education. However, the nurses acknowledged information and education needs to be shared beyond women receiving services at the facility.

Study participants often discussed the need for improved messaging targeting men. Previous research has stated that men lack the opportunity to be taught about HIV/AIDS and ART, as women may utilize health facilities more than men because of pregnancy [19]. In this study, both the women and nurses discussed Swazi men lacking information and education about HIV.

Nurses recommended engaging men by providing HIV testing at locations that men frequent. A review of male involvement initiatives in PMTCT provided a few recommendations including restructuring maternal health clinics so that they are culturally and practically acceptable to men, changing social and behavioral norms through use of peer programs and community leaders and adding outreach programs such as providing HIV testing at home, personal invitations and other approaches, but results are very mixed across country settings [25].

Study participants also discussed that Swazi men have strong fears about HIV testing and claimed that some men even discourage their partners from receiving ANC services [26], as some men assume that their partner’s positive status is an indication of their own HIV status [27, 28]. One study noted that some women experienced direct discouragement from their partner and that women who did not live with their boyfriends had more support to join a PMTCT program [24] which could perhaps be attributed to stigma and fear of ARVs being seen in the house. In a study in Lesotho some nurses claimed that fear of disclosing to the partner is the main obstacle, as the men will then blame the woman for bringing HIV into the house [29].

With Option B+ significantly increasing the number of women that will begin lifelong ART, it will become even more important to engage the community, especially men about the importance of lifelong ART to ensure women receive support to initiate and adhere to ART. The nurses recommend that men receive information about HIV and ART at the locations they frequent such as “dip tanks”, churches and community meetings. Other organizations in Swaziland are currently informing and educating men about HIV at their workplaces (especially in the urban areas). However, more information is needed about where is the best place to provide educational messages to men, who would be the best persons to provide these education messages to men, which messages are most effective with men and where is the best place for men to receive HIV tests.

### Strengths and limitations

One of the strengths of this study was the use of two data collection methods (interviews and FGDs) to understand both the individual experience and the general perspective of pregnant women and nurses. One limitation was interviews being conducted by nurse midwives who may have been involved in the care of study participants. Another limitation was having only one FGD with women who did not initiate ART; this was a result of challenges recruiting this population.

## Conclusion

As Option B+ continues to be rolled-out across Swaziland, an increasing number of women who may be asymptomatic and feeling “healthy” will be asked to initiate lifelong ART. Both women and nurses discussed major concerns about the commitment of  lifelong ART. The findings highlight the need for strengthened messaging about the benefits of lifelong ART, specifically to the mother and her partner.

In addition, there is an increased need for information sharing with the community about the current drugs being used in ART, and specifically informing the public that the drug which caused lipodystrophy deformities is no longer in use. As patients tend to have many questions regarding drug side effects, nurses and other health staff should discuss the side effects promptly with the patients to alleviate their inaccurate perceptions. 

One method proposed by the nurses to decrease LTFU is allowing increased flexibility for nurses to determine the number of adherence counseling sessions a specific woman receives. Creating a flexible system in which nurses can identify those who need additional counseling could enable nurses to keep women at higher risk of LTFU within the system. Implementing and improving systems that follow mother-baby pairs through the risk period for MTCT and tracking those who do not return to the facility has become increasingly important under Option B+ [30].

To address the information gap among men about HIV and ART, study participants cited the importance of targeted messaging and knowledge improvement, particularly around disclosure and status. As HIV and ART are still stigmatized topics, women are less likely to discuss them with their partners. It is well documented in the literature that disclosure is a significant challenge and directly affects adherence. Finding ways of accessing and educating men in the community about HIV and ART will be critical for Option B+ to be effective.

The findings from this study highlight the challenges that women have accepting lifelong ART. Previous research has explored barriers initiating PMTCT and ART but little research has sought to understand the concerns associated directly with ART being a lifelong commitment. Understanding what women are concerned about will aid in developing effective counseling messages, designing appropriate counseling structures, understanding where additional support is needed, and knowing where to target community level messages.

## Abbreviations

AIDS: Acquired immunodeficiency syndrome
ANC: Antenatal care
ART: Antiretroviral therapy
ARV: Antiretroviral drug
CD4: Cluster of differentiation 4
EGPAF: Elizabeth Glaser Pediatric AIDS Foundation
FGD: Focus group discussion
HIV: Human immunodeficiency virus
LTFU: Lost to follow-up
MTCT: Mother-to-child transmission
PMTCT: Prevention of mother-to-child transmission
PNC: Post-natal care
WHO: World Health Organization

## Footnotes

1 Commonly used in Swaziland to describe short-course prophylaxis given during Option A.

## References

[1] Nolan ML, Greenberg AE, Fowler MG (2002). A review of clinical trials to prevent mother-to-child HIV-1 transmission in Africa and inform rational intervention strategies. Aids.

[2] Kim MH (2012). The Tingathe programme: a pilot intervention using community health workers to create a continuum of care in the prevention of mother to child transmission of HIV (PMTCT) cascade of services in Malawi. J Int AIDS Soc.

[3] Health, MO. 12th National HIV Serosurveillance among women attending antenatal care services in Swaziland. Mbabane, Swaziland; 2010

[4] Central Statistical Office (CSO) [Swaziland], a.M.I.I (2008). Swaziland demographic and health survey 2006–07.

[5] WHO (2010). WHO guidelines approved by the guidelines review committee. Antiretroviral drugs for treating pregnant women and preventing HIV infection in infants: recommendations for a public health approach: 2010 version.

[6] Organization, WH (2013). Consolidated guidelines on the use of antiretroviral drugs for treating and preventing HIV infection.

[7] Adler MR, CC, Sundaram M, Lukhele B, Vandelanotte J, Mwanyumba F. Using Swaziland’s national early infant diagnosis database to inform PMTCT program interventions. Proceedings of the XVIII International AIDS Conference; 2010 July 18–23; Vienna, Austria.

[8] Health, SMO (2013). Annual HIV programs report.

[9] Assefa Y (2010). Toward universal access to HIV counseling and testing and antiretroviral treatment in Ethiopia: looking beyond HIV testing and ART initiation. AIDS Patient Care STDs.

[10] Fox MP (2010). Barriers to initiation of antiretroviral treatment in rural and urban areas of Zambia: a cross-sectional study of cost, stigma, and perceptions about ART. J Int AIDS Soc.

[11] Gourlay A (2013). Barriers and facilitating factors to the uptake of antiretroviral drugs for prevention of mother-to-child transmission of HIV in sub-Saharan Africa: a systematic review. J Int AIDS Soc.

[12] Govindasamy D, Ford N, Kranzer K (2012). Risk factors, barriers and facilitators for linkage to antiretroviral therapy care: a systematic review. Aids.

[13] Johnson DC (2013). Factors associated with timely initiation of antiretroviral therapy in two HIV clinics in Lilongwe, Malawi. Int J STD AIDS.

[14] Vaismoradi M, Turunen H, Bondas T (2013). Content analysis and thematic analysis: implications for conducting a qualitative descriptive study. Nurs Health Sci.

[15] Parker LA (2015). Implementation and operational research: barriers and facilitators to combined ART initiation in pregnant women with HIV: lessons learnt from a PMTCT B+ pilot program in Swaziland. J Acquir Immune Defic Syndr.

[16] Tenthani L (2014). Retention in care under universal antiretroviral therapy for HIV-infected pregnant and breastfeeding women (‘Option B+’) in Malawi. Aids.

[17] Black S, et al. Acceptability and challenges of rapid ART initiation among pregnant women in a pilot programme, Cape Town, South Africa. AIDS Care. 2014;26(6):736–41. doi:10.1080/09540121.2013.855300. Epub 2013 Nov 7. 10.1080/09540121.2013.855300 24200029

[18] Kahn TR (2013). Delayed initiation of antiretroviral therapy among HIV-discordant couples in Kenya. AIDS Care.

[19] Duff P (2010). Barriers to accessing highly active antiretroviral therapy by HIV-positive women attending an antenatal clinic in a regional hospital in western Uganda. J Int AIDS Soc.

[20] Musheke M, Bond V, Merten S (2013). Deterrents to HIV-patient initiation of antiretroviral therapy in urban Lusaka, Zambia: a qualitative study. AIDS Patient Care STDs.

[21] Iroezi ND (2013). A qualitative analysis of the barriers and facilitators to receiving care in a prevention of mother-to-child program in Nkhoma, Malawi. Afr J Reprod Health.

[22] Stinson K, Myer L (2012). Barriers to initiating antiretroviral therapy during pregnancy: a qualitative study of women attending services in Cape Town, South Africa. Afr J AIDS Res.

[23] Curran K (2014). ‘If I am given antiretrovirals I will think I am nearing the grave’: Kenyan HIV serodiscordant couples’ attitudes regarding early initiation of antiretroviral therapy. Aids.

[24] Eide M (2006). Social consequences of HIV-positive women’s participation in prevention of mother-to-child transmission programmes. Patient Educ Couns.

[25] Dunlap J (2014). Male involvement for the prevention of mother-to-child HIV transmission: a brief review of initiatives in east, west, and central Africa. Curr HIV/AIDS Rep.

[26] Nyasulu JYNAP (2011). Barriers to the upltake of prevention of mother to child transmission (PMTCT) services in rural Blantyre and Balaka districts in Malawi. Journal of Rural and Tropical Public Health.

[27] Chinkonde JR, Sundby J, Martinson F (2009). The prevention of mother-to-child HIV transmission programme in Lilongwe, Malawi: why do so many women drop out. Reprod Health Matters.

[28] Falnes EF (2011). “It is her responsibility”: partner involvement in prevention of mother to child transmission of HIV programmes, northern Tanzania. J Int AIDS Soc.

[29] Towle M, Lende DH (2008). Community approaches to preventing mother-to-child HIV transmission: perspectives from rural Lesotho. Afr J AIDS Res.

[30] Kieffer MP (2014). Lessons learned from early implementation of option B+: the Elizabeth Glaser Pediatric AIDS Foundation experience in 11 African countries. J Acquir Immune Defic Syndr.

